# Benzene-1,4-dicarboxylic acid–*N*,*N*-dimethyl­acetamide (1/2)

**DOI:** 10.1107/S1600536809025793

**Published:** 2009-07-08

**Authors:** Xia Guo, Youwei Cheng, Xi Li

**Affiliations:** a College of Materials Science and Chemical Engineering, Zhejiang University, Zhejiang 310027, People’s Republic of China

## Abstract

The asymmetric unit of title compound, C_8_H_6_O_4_·2C_4_H_9_NO, contains one half-mol­ecule (an inversion centre in *P*21/*n* generates the other half of the molecule) of terephthalic acid (TA) and one mol­ecule of *N*,*N*-dimethyl­acetamide (DMAC). The DMAC mol­ecules are linked to TA by strong O—H⋯O hydrogen bonds.

## Related literature

For the crystal structure of terephthalic acid-bis­(*N*,*N*-dimethyl­formamide), see: Dale & Elsegood (2004 ▶). For the polymorphism of terephthalic acid, see: Bailey & Brown (1967 ▶); Sledz *et al.* (2001 ▶).
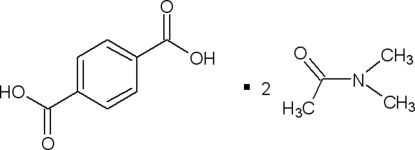


## Experimental

### 

#### Crystal data

C_8_H_6_O_4_·2C_4_H_9_NO
*M* *_r_* = 340.37
Monoclinic, 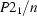

*a* = 10.191 (2) Å
*b* = 8.5228 (17) Å
*c* = 10.719 (2) Å
β = 110.67 (3)°
*V* = 871.0 (3) Å^3^
*Z* = 2
Mo *K*α radiation
μ = 0.10 mm^−1^
*T* = 113 K
0.60 × 0.51 × 0.38 mm

#### Data collection

Rigaku R-AXIS RAPID diffractometer
Absorption correction: multi-scan (*ABSCOR*; Higashi, 1995 ▶) *T*_min_ = 0.940, *T*_max_ = 0.961
6605 measured reflections
1522 independent reflections
1395 reflections with *I* > 2σ(*I*)
*R*_int_ = 0.014

#### Refinement

*R*[*F* ^2^ > 2σ(*F* ^2^)] = 0.043
*wR*(*F* ^2^) = 0.118
*S* = 1.08
1522 reflections
116 parameters
H atoms treated by a mixture of independent and constrained refinement
Δρ_max_ = 0.58 e Å^−3^
Δρ_min_ = −0.26 e Å^−3^

### 

Data collection: *RAPID-AUTO* (Rigaku, 1998 ▶); cell refinement: *RAPID-AUTO*; data reduction: *RAPID-AUTO*; program(s) used to solve structure: *SHELXS97* (Sheldrick, 2008 ▶); program(s) used to refine structure: *SHELXL97* (Sheldrick, 2008 ▶); molecular graphics: *SHELXTL* (Sheldrick, 2008 ▶); software used to prepare material for publication: *SHELXL97*.

## Supplementary Material

Crystal structure: contains datablocks I, global. DOI: 10.1107/S1600536809025793/gk2216sup1.cif

Structure factors: contains datablocks I. DOI: 10.1107/S1600536809025793/gk2216Isup2.hkl

Additional supplementary materials:  crystallographic information; 3D view; checkCIF report

## Figures and Tables

**Table 1:** Hydrogen-bond geometry (Å, °)

*D*—H⋯*A*	*D*—H	H⋯*A*	*D*⋯*A*	*D*—H⋯*A*
O2—H2⋯O1^i^	0.91 (3)	1.65 (3)	2.551 (2)	173 (2)

Symmetry code: (i) 
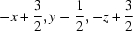
.

**Fig. 1.**
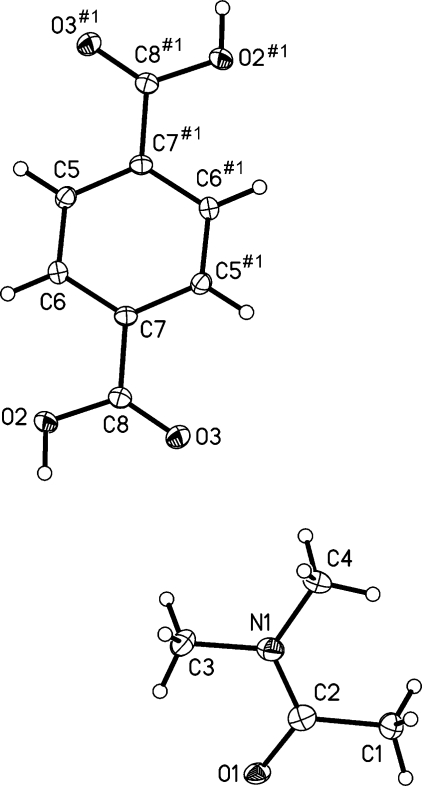
View of the title compound showing 50% probability displacement ellipsoids. Symmetry operation for atoms with '#': -x,-y,-z + 1.

**Fig. 2.**
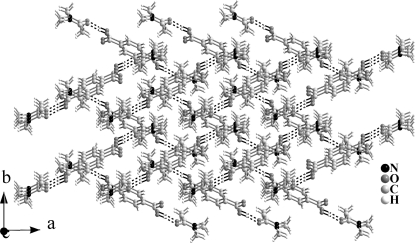
The packing diagram for the title compound; dashed lines indicate hydrogen bonds.

## supplementary crystallographic information

### Comment

Terephthalic acid (TA) is an important intermediate in the production of polyesters for plastics and fiber applications. According to Bailey & Brown (1967), TA exists in two polymorphic modifications (forms 1 and 2), both triclinic. Recently Sledz *et al.* (2001) reported a new crystalline form of TA which is monoclinic and designated as form 3.

*N*,*N*-Dimethylformamide (DMF) and *N*,*N*-dimethylacetamide (DMAC) are the two of a few organic solvents capable of dissolving TA. The crystal structure of the 2:1 DMF solvate of terephthalic acid was reported recently (Dale & Elsegood, 2004). The solvent molecules and TA form a centrosymmetric descrete planar assembly with both carboxylic acid groups hydrogen bonded to DMF molecules *via R*_2_^2^(7) motif (O—H···O/C—H···O interactions). Recently we have obtained single crystals of the DMAC solvate of TA and here we report its crystal structure.

The asymmetric unit of title compound contains one half-molecule of TA and one N, *N*-dimethyl acetamide (DMAC) molecule (Fig. 2). The DMAC molecules are linked to TA by strong O—H···O hydrogen bonds (Fig.3 and Table 1), which may be effective in stablilizing the crystal structure. The carboxylic group is roughly coplanar with the benzene ring forming dihedral angle of 0.6 (3)° . The dihedral angle between TA and the dimethylacetamide molecule is 21.7 (1)°.

### Experimental

Single crystals were obtained by dissolving TA (1.0 g) in DMAC (20 ml) at 80°C and then allowing the solvent to cool to room temperature. The sample proved unstable in the air.

### Refinement

The H atom of the carboxylic group was located from a difference Fourier map and fully refined. The remaining H atoms were placed in geometrically calculated positions and refined using a riding model, with *U*_iso_(H) = 1.2 *U*_eq_(C).

### Crystal data

**Table:**

C_8_H_6_O_4_·2C_4_H_9_NO	*F*(000) = 364
*M**_r_* = 340.37	*D*_x_ = 1.298 Mg m^−^^3^
Monoclinic, *P*2_1_/*n*	Mo *K*α radiation, λ = 0.71073 Å
Hall symbol: -P 2yn	Cell parameters from 6873 reflections
*a* = 10.191 (2) Å	θ = 3.1–27.5°
*b* = 8.5228 (17) Å	µ = 0.10 mm^−^^1^
*c* = 10.719 (2) Å	*T* = 113 K
β = 110.67 (3)°	Block, colorless
*V* = 871.0 (3) Å^3^	0.60 × 0.51 × 0.38 mm
*Z* = 2	

### Data collection

**Table:**

Rigaku R-AXIS RAPID diffractometer	1522 independent reflections
Radiation source: fine-focus sealed tube	1395 reflections with *I* > 2σ(*I*)
graphite	*R*_int_ = 0.014
Detector resolution: 0 pixels mm^-1^	θ_max_ = 25.0°, θ_min_ = 3.1°
ω scans	*h* = −11→11
Absorption correction: multi-scan (*ABSCOR*; Higashi, 1995)	*k* = −9→10
*T*_min_ = 0.940, *T*_max_ = 0.961	*l* = −12→12
6605 measured reflections	

### Refinement

**Table:**

Refinement on *F*^2^	Primary atom site location: structure-invariant direct methods
Least-squares matrix: full	Secondary atom site location: difference Fourier map
*R*[*F*^2^ > 2σ(*F*^2^)] = 0.043	Hydrogen site location: inferred from neighbouring sites
*wR*(*F*^2^) = 0.118	H atoms treated by a mixture of independent and constrained refinement
*S* = 1.08	*w* = 1/[σ^2^(*F*_o_^2^) + (0.0571*P*)^2^ + 0.6867*P*] where *P* = (*F*_o_^2^ + 2*F*_c_^2^)/3
1522 reflections	(Δ/σ)_max_ < 0.001
116 parameters	Δρ_max_ = 0.58 e Å^−^^3^
0 restraints	Δρ_min_ = −0.26 e Å^−^^3^

### Special details

**Table:**

Geometry. All e.s.d.'s (except the e.s.d. in the dihedral angle between two l.s. planes) are estimated using the full covariance matrix. The cell e.s.d.'s are taken into account individually in the estimation of e.s.d.'s in distances, angles and torsion angles; correlations between e.s.d.'s in cell parameters are only used when they are defined by crystal symmetry. An approximate (isotropic) treatment of cell e.s.d.'s is used for estimating e.s.d.'s involving l.s. planes.
Refinement. Refinement of *F*^2^ against ALL reflections. The weighted *R*-factor *wR* and goodness of fit *S* are based on *F*^2^, conventional *R*-factors *R* are based on *F*, with *F* set to zero for negative *F*^2^. The threshold expression of *F*^2^ > σ(*F*^2^) is used only for calculating *R*-factors(gt) *etc*. and is not relevant to the choice of reflections for refinement. *R*-factors based on *F*^2^ are statistically about twice as large as those based on *F*, and *R*- factors based on ALL data will be even larger.

### Fractional atomic coordinates and isotropic or equivalent isotropic displacement parameters (Å^2^)

**Table:**

	*x*	*y*	*z*	*U*_iso_*/*U*_eq_	
N1	0.74204 (15)	0.07465 (18)	0.88639 (15)	0.0233 (4)	
O1	0.95506 (11)	0.13078 (14)	0.88125 (11)	0.0207 (3)	
O2	0.30574 (13)	−0.23616 (15)	0.52621 (12)	0.0248 (3)	
O3	0.35578 (12)	−0.10972 (15)	0.72025 (11)	0.0237 (3)	
C1	0.89721 (19)	0.2372 (2)	1.06449 (18)	0.0266 (4)	
H1A	0.9097	0.1670	1.1378	0.040*	
H1B	0.8203	0.3067	1.0557	0.040*	
H1C	0.9813	0.2973	1.0806	0.040*	
C2	0.86637 (18)	0.1442 (2)	0.93832 (17)	0.0224 (4)	
C3	0.71443 (18)	−0.0191 (2)	0.76458 (17)	0.0241 (4)	
H3A	0.7248	0.0458	0.6954	0.036*	
H3B	0.6206	−0.0598	0.7363	0.036*	
H3C	0.7799	−0.1046	0.7824	0.036*	
C4	0.63337 (19)	0.0830 (3)	0.9455 (2)	0.0304 (5)	
H4A	0.6764	0.0888	1.0408	0.046*	
H4B	0.5755	−0.0090	0.9216	0.046*	
H4C	0.5768	0.1746	0.9131	0.046*	
C5	−0.08828 (17)	−0.03522 (19)	0.37140 (16)	0.0171 (4)	
H5	−0.1477	−0.0586	0.2852	0.020*	
C6	0.04270 (17)	−0.10394 (19)	0.42154 (16)	0.0175 (4)	
H6A	0.0712	−0.1734	0.3692	0.021*	
C7	0.13228 (16)	−0.06918 (18)	0.55081 (15)	0.0152 (4)	
C8	0.27579 (16)	−0.13973 (18)	0.60876 (15)	0.0163 (4)	
H2	0.394 (3)	−0.276 (3)	0.563 (2)	0.052 (7)*	

### Atomic displacement parameters (Å^2^)

**Table:**

	*U*^11^	*U*^22^	*U*^33^	*U*^12^	*U*^13^	*U*^23^
N1	0.0191 (8)	0.0254 (8)	0.0268 (8)	−0.0007 (6)	0.0097 (6)	0.0006 (6)
O1	0.0166 (6)	0.0255 (7)	0.0209 (6)	−0.0043 (5)	0.0080 (5)	0.0014 (5)
O2	0.0174 (7)	0.0324 (7)	0.0224 (7)	0.0083 (5)	0.0044 (5)	−0.0055 (5)
O3	0.0182 (6)	0.0282 (7)	0.0200 (6)	0.0047 (5)	0.0012 (5)	−0.0035 (5)
C1	0.0258 (9)	0.0256 (10)	0.0299 (10)	−0.0037 (7)	0.0117 (8)	−0.0079 (7)
C2	0.0227 (9)	0.0179 (9)	0.0248 (9)	0.0013 (7)	0.0060 (7)	0.0051 (7)
C3	0.0227 (9)	0.0265 (10)	0.0205 (9)	−0.0061 (7)	0.0047 (7)	−0.0023 (7)
C4	0.0192 (9)	0.0407 (11)	0.0344 (11)	−0.0024 (8)	0.0133 (8)	−0.0086 (8)
C5	0.0168 (8)	0.0189 (8)	0.0145 (8)	−0.0012 (6)	0.0043 (6)	−0.0011 (6)
C6	0.0187 (8)	0.0176 (8)	0.0171 (8)	0.0005 (6)	0.0076 (6)	−0.0018 (6)
C7	0.0140 (8)	0.0156 (8)	0.0170 (8)	−0.0014 (6)	0.0066 (6)	0.0022 (6)
C8	0.0164 (8)	0.0159 (8)	0.0175 (8)	−0.0009 (6)	0.0072 (6)	0.0017 (6)

### Geometric parameters (Å, °)

**Table:**

N1—C2	1.330 (2)	C3—H3B	0.9600
N1—C4	1.459 (2)	C3—H3C	0.9600
N1—C3	1.471 (2)	C4—H4A	0.9600
O1—C2	1.263 (2)	C4—H4B	0.9600
O2—C8	1.320 (2)	C4—H4C	0.9600
O2—H2	0.91 (3)	C5—C6	1.381 (2)
O3—C8	1.213 (2)	C5—C7^i^	1.397 (2)
C1—C2	1.502 (2)	C5—H5	0.9300
C1—H1A	0.9600	C6—C7	1.396 (2)
C1—H1B	0.9600	C6—H6A	0.9300
C1—H1C	0.9600	C7—C5^i^	1.397 (2)
C3—H3A	0.9600	C7—C8	1.498 (2)
			
C2—N1—C4	123.61 (16)	N1—C4—H4A	109.5
C2—N1—C3	117.98 (15)	N1—C4—H4B	109.5
C4—N1—C3	118.39 (14)	H4A—C4—H4B	109.5
C8—O2—H2	111.4 (16)	N1—C4—H4C	109.5
C2—C1—H1A	109.5	H4A—C4—H4C	109.5
C2—C1—H1B	109.5	H4B—C4—H4C	109.5
H1A—C1—H1B	109.5	C6—C5—C7^i^	120.51 (15)
C2—C1—H1C	109.5	C6—C5—H5	119.7
H1A—C1—H1C	109.5	C7^i^—C5—H5	119.7
H1B—C1—H1C	109.5	C5—C6—C7	119.99 (15)
O1—C2—N1	119.73 (16)	C5—C6—H6A	120.0
O1—C2—C1	121.74 (15)	C7—C6—H6A	120.0
N1—C2—C1	118.53 (16)	C6—C7—C5^i^	119.50 (15)
N1—C3—H3A	109.5	C6—C7—C8	121.80 (15)
N1—C3—H3B	109.5	C5^i^—C7—C8	118.70 (14)
H3A—C3—H3B	109.5	O3—C8—O2	123.91 (15)
N1—C3—H3C	109.5	O3—C8—C7	122.83 (15)
H3A—C3—H3C	109.5	O2—C8—C7	113.26 (14)
H3B—C3—H3C	109.5		

Symmetry codes: (i) −*x*, −*y*, −*z*+1.

### Hydrogen-bond geometry (Å, °)

**Table:**

*D*—H···*A*	*D*—H	H···*A*	*D*···*A*	*D*—H···*A*
O2—H2···O1^ii^	0.91 (3)	1.65 (3)	2.551 (2)	173 (2)

Symmetry codes: (ii) −*x*+3/2, *y*−1/2, −*z*+3/2.

## References

[bb1] Bailey, M. & Brown, C. J. (1967). *Acta Cryst.* **22**, 387–391.

[bb2] Dale, S. H. & Elsegood, M. R. J. (2004). *Acta Cryst.* C**60**, o444–o448. 10.1107/S010827010400976X 15178875

[bb3] Higashi, T. (1995). *ABSCOR* Rigaku Corporation, Tokyo, Japan.

[bb4] Rigaku (1998). *RAPID-AUTO* Rigaku Corporation, Tokyo, Japan.

[bb5] Sheldrick, G. M. (2008). *Acta Crtyst.* A**64**, 112-122.

[bb6] Sledz, M., Janczak, J. & Kubiak, R. (2001). *J. Mol. Struct.* 595, 77–82.

